# Climatic factors and the incidence of dengue in Cartagena, Colombian Caribbean Region

**DOI:** 10.1590/0037-8682-0072-2022

**Published:** 2022-09-30

**Authors:** Eder Cano-Pérez, Steev Loyola, Dacia Malambo-García, Doris Gómez-Camargo

**Affiliations:** 1 Universidad de Cartagena, Facultad de Medicina, Grupo de Investigación UNIMOL, Cartagena de Indias, Colombia.; 2 Universidad de Cartagena, Facultad de Medicina, Programa de Doctorado en Medicina Tropical, Cartagena de Indias, Colombia.; 3 Universidad Peruana Cayetano Heredia, Facultad de Medicina, Lima, Peru.

**Keywords:** Dengue, Mosquito-borne disease, Climatic factors, Macroclimatic phenomena, Colombian Caribbean region

## Abstract

**Background:**

The influence of climate on the epidemiology of dengue has scarcely been studied in Cartagena.

**Methods:**

The relationship between dengue cases and climatic and macroclimatic factors was explored using an ecological design and bivariate and time-series analyses during lag and non-lag months. Data from 2008-2017 was obtained from the national surveillance system and meteorological stations.

**Results:**

Cases correlated only with climatic variables during lag and non-lag months. Decreases in precipitation and humidity and increases in temperature were correlated with an increase in cases.

**Conclusions:**

Our findings provide useful information for establishing and strengthening dengue prevention and control strategies.

*Aedes aegypti*, the main vector of arboviruses such as dengue, *Zika*, and *Chikungunya*, is widely distributed in Colombia^1^. *Chikungunya* and *Zika* were first detected in Colombia in 2013 and 2016, respectively. Both arboviruses are widespread throughout the country, causing large outbreaks and thousands of confirmed cases^1^. However, compared to the dengue virus, *Zika* and *Chikungunya* virus transmission is not long-lasting. In Colombia, dengue is hyperendemic and has sustained transmission, causing an average of 85000 cases per year^1^. Globally, dengue incidence and transmission are influenced by the interaction of sociodemographic, environmental, ecological, and weather-related factors^1^. 

In the last decade, much research in the Americas and around the world has focused on the relationship between weather factors (climatic and macroclimatic) and dengue incidence and transmission. However, this relationship was not consistent or comparable between and within the tropical regions of Latin America surveyed. In Mexico, the El Niño phenomenon and increased temperatures were described as factors correlated with an increase in dengue cases, whereas precipitation has been described as a non-influential factor^2^. In contrast, in Trinidad and Tobago and Honduras, increased precipitation correlated with an increase in cases; interestingly, the temperature did not appear to be a factor related to dengue case incidence^2^. In Honduras, La Niña correlated with an increase in the number of cases. These discrepant results, even for countries in the same region, could be explained by different geographic, socioeconomic, or cultural characteristics^2^.

In Colombia, dengue cases were negatively correlated with precipitation and positively correlated with the temperature at the national level. Furthermore, the El Niño phenomenon correlated with increased cases in the country^3^. However, these correlations may vary at lower spatial scales, such as regional, departmental, and municipal scales, and extrapolation of relationships between climate variables or phenomena and the number of dengue cases from one scale to another would be inappropriate without prior evaluation^3^. In the Caribbean region of Colombia, there are several tropical dengue-endemic areas; however, the relationship between dengue incidence and weather factors has hardly been described in the municipalities of this region^4^. The municipality of Cartagena de Indias is the capital of the Bolívar Department **(** Figure 1 **)** and one of the most important urbanized cities in the Colombian Caribbean region due to its economic, industrial, and tourist activities. In Cartagena, dengue has been classified as a public health threat due to its endemicity and high mortality rate, which exceeded the national rate for the last decade^5^. However, to the best of our knowledge, the relationships between weather variables and dengue dynamics in Cartagena have not been analyzed. Therefore, in the context of evidence-based decision-making, this lack of information translates into a gap that limits the development and strengthening of dengue prevention and control strategies in Cartagena. Herein, we describe the relationships between documented dengue cases and weather variables for the period 2008-2017 in the municipality of Cartagena de Indias, Colombia. 

**FIGURE 1:**
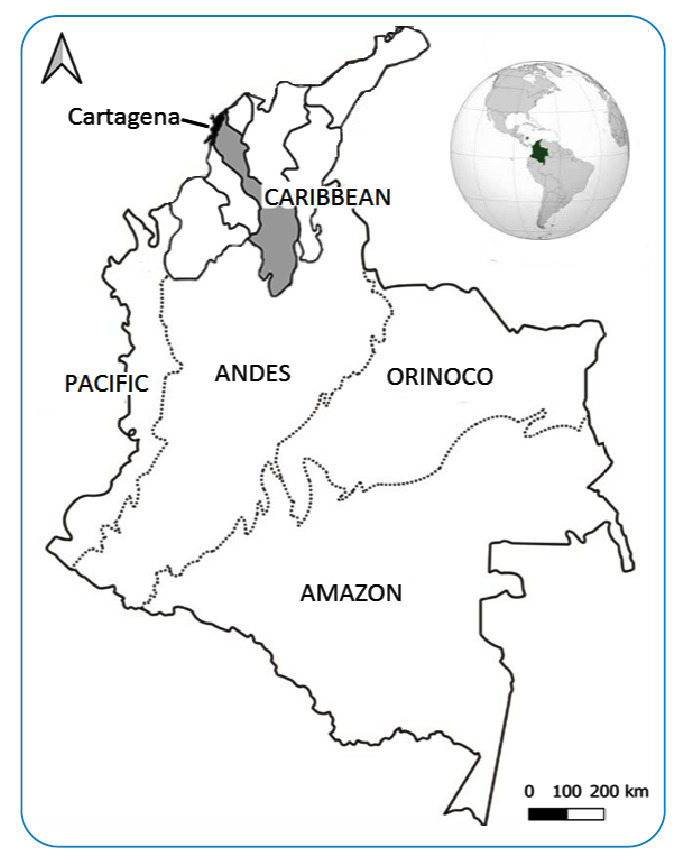
Location of the study area, Cartagena, Colombia.

Laboratory-confirmed dengue cases were obtained from the National Public Health Surveillance System (SIVIGILA) of the Colombian National Institute of Health (http://portalsivigila.ins.gov.co/Paginas/Vigilancia-Rutinaria.aspx). The information collected included documented cases from the first epidemiological week in 2008 to the last epidemiological week in 2017. The information was aggregated at the monthly level based on each epidemiological week. In addition, information on monthly precipitation and rainy days, and monthly averages of humidity and temperature, was obtained from the meteorological station of the Oceanographic and Hydrographic Research Center (Centro de Investigaciones Oceanográficas e hidrográficas) in Cartagena (www.cioh.org.co/meteorologia/wrf.php?t=lt&dom=d02). El Niño and La Niña macroclimatic phenomena were determined by the Oceanic Niño Index (ONI) and the Southern Oscillation Index (SOI) based on National Oceanic and Atmospheric Administration (NOAA) criteria^6^. ONI values of -0.5 or lower and positive SOI values were used to define the La Niña period (period of abundant precipitation in Colombia), while the El Niño (period of no or little precipitation in Colombia) was defined by ONI values of 0.5 or higher and negative SOI values, as described elsewhere^6^. Since there are no threshold values for SOI, the Neutral period was defined only with ONI values between 0.5 and -0.5. Time series graphs were plotted using case data and climatic and macroclimatic variables. Correlation analyses were then performed using Spearman's test for the month of co-occurrence (month 0) and 12-month lags. Differences in the number of cases by ONI and SOI periods were explored using the Kruskal-Wallis test and the Mann-Whitney U test, respectively. All analyses were performed in SPSS v.19.0 (IBM Corp, Armonk, NY) and Stata v.16.1 (StataCorp. 2019. Stata Statistical Software: Release 16. College Station, TX: StataCorp LLC.).

During the study period, 5223 laboratory-confirmed dengue cases were reported in Cartagena, with months in which at least one case was reported and others with a maximum of 365 cases. The median monthly and an annual number of cases was 15 (interquartile range [IQR] = 45.5) and 271 (IQR = 637), respectively. The monthly incidence of dengue cases was comparable between February and December (Figure 2, panel A), with January having the highest number of reported cases (16.6%, 867/5223). On an annual basis, 44.5% (2326/5223) of cases were reported in 2013, the year with the highest recorded transmission (Figure 2, panel B). 

**FIGURE 2:**
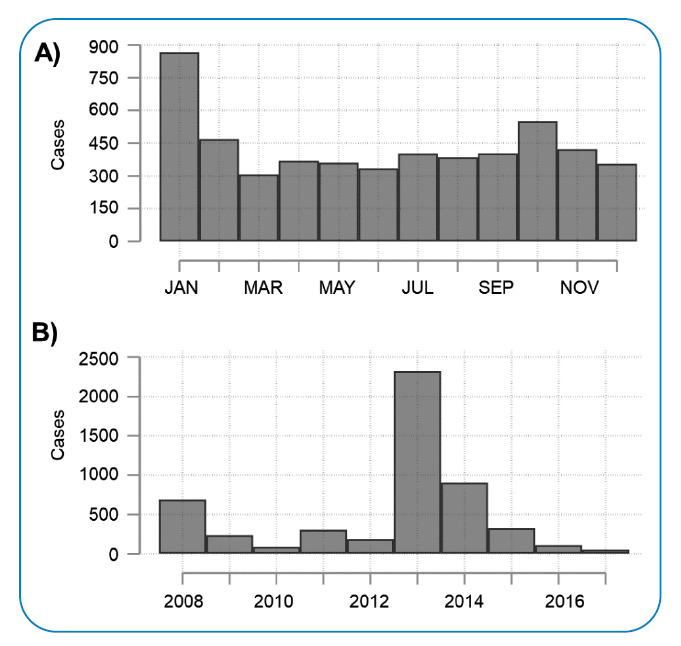
Annual **(A)** and monthly **(B)** frequency of dengue cases in Cartagena, Colombia, between 2008 and 2017.

Time series and bivariate analyses of dengue cases and weather factors during the study period are shown in Figure 3. The average temperature (coefficient of variation (CV) = 2.8%, Figure 3, panel A) and humidity (CV = 6.2%, Figure 3, panel C) exhibited low variability compared with rainy days (CV = 92.1%, Figure 3, panel E) and precipitation (CV = 118.2%, Figure 3, panel G). As expected, these two climatic variables had similar patterns (Figure 3, panels E and G). The increase in temperature was correlated with the increase in cases between the first and eighth lagged months (Figure 3, panel B), whereas humidity and cases were negatively correlated in the non-lagged and first lagged months (Figure 3, panel D). Cumulative precipitation (Figure 3, panel F) and rainy days (Figure 3, panel H) were negatively correlated with cases between the 7^th^ and 10^th^ and 6^th^ and 12^th^ lagged months, respectively. Remarkably, ONI and SOI did not correlate with dengue cases (Figure 3, panels I through L), and no differences were observed between reported cases for El Niño-Southern Oscillation (ENSO) phases defined by ONI (p = 0.177; Kruskal-Wallis) or SOI (p = 0.370; Mann-Whitney U).

**FIGURE 3:**
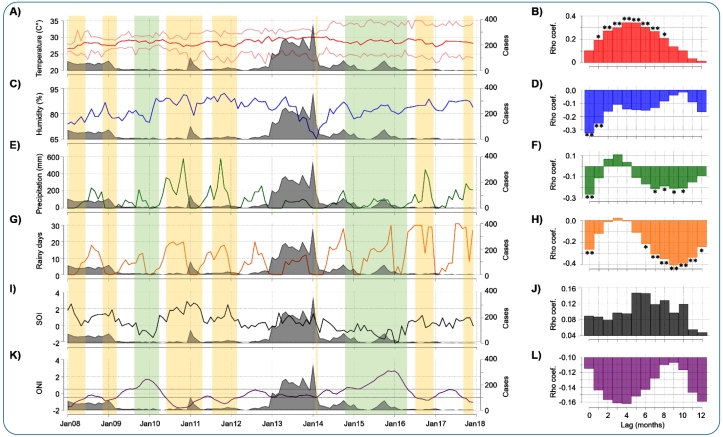
Time-series plots and bivariate cross-correlations between dengue cases and average temperature **(A, B)**, relative humidity **(C, D)**, precipitation **(E, F)**, rainy days **(G, H)**, SOI **(I, J)**, and ONI **(K, L)** in Cartagena. In the time-series plots, the vertical green stripes correspond to El Niño periods, yellow stripes to La Niña periods, and white stripes correspond to Neutral periods. In the correlation graphs, an asterisk (*) denotes a p-value <0.050, while two asterisks (**) denote a p-value <0.001.

Our results suggest that macroclimatic phenomena do not play a role in dengue dynamics in Cartagena. These findings are consistent with those previously reported in the Caribbean region and Bolívar Department, where no significant correlations between dengue cases, macroclimatic phenomena, and climatic variables were found^3^. However, in contrast to the observations at these higher geographic scales, climatic variables and dengue cases were correlated in Cartagena. In particular, the decrease in precipitation and humidity and the increase in temperature were correlated with the increase in dengue cases. Our findings are similar to those previously reported for other Colombian municipalities in the Caribbean^4^. Overall, a positive relationship between dengue cases, rainy days, and precipitation is expected, as increased rainfall creates more breeding sites for mosquitoes and larval development^7^. However, it is possible that, as in other Colombian areas, warm and dry conditions interact with social-ecological, biological, and entomological factors to play an important role in the incidence of dengue cases in Cartagena^3^. 

Various conditions contribute to the establishment of mosquito breeding sites in urban areas. The temperature and its low fluctuation, both observed throughout the study period, may have favored the survival and rapid development of *Ae. aegypti*, as well as the efficient transmission of dengue virus due to the high viral replication among vectors and reduced incubation period^7^. In addition, deficiencies in the public water supply and the resulting need to store water in containers for long periods are factors that favor breeding sites in urban environments. These factors have been previously described in Cartagena^8^ and certainly favor the sustainability of breeding sites since water containers often do not have lids and are exposed to the environment. In contrast, it is conceivable that the breeding sites could be disrupted by heavy rainfall lasting several days, leading to a decline in vector populations and an immediate and lagged negative impact on dengue transmission in Cartagena^9^. However, the lagged effects of rainfall and rainy days on dengue cases (Figure 3, panels F and H) may not be explained solely by the alteration of breeding sites; there could also be other interactions with other factors not analyzed here or factors that are not yet clearly understood. Further studies are required to confirm the consistency of our findings.

In Colombia, previous studies conducted in two municipalities in the Andean region^10^ ^,^ ^11^ and two other municipalities in the Caribbean region^12^ ^,^ ^13^ have suggested that increases in dengue cases correlate with increases in precipitation. However, the discrepancy between these findings and those reported here could be explained by different transmission patterns related to major determinants such as social and economic factors, urbanization, geographic expansion of vectors, and human mobilization^1^ that can alter dengue incidence between regions and even between municipalities within the same region. Therefore, the discrepancy in results may reflect the need to provide evidence at the local level and may serve as a warning not to extrapolate associations, even between contiguous localities.

The largest dengue outbreak in Colombia in the last 42 years was observed in 2010^14^. Approximately 80.0% of the cases reported in 2010 were detected in the Andean and Pacific regions, while 8.5% were detected in the Caribbean region. However, during the 2013 outbreak in Colombia, the Caribbean was the second region with the highest number of dengue cases, contributing to approximately 30.5% of the total^14^. In Cartagena, no significant increase in the number of cases was detected in 2010. However, a significant increase in the number of cases was observed in 2013 (Figure 2A), similar to that previously described^14^. 

Our findings must be interpreted cautiously, as the correlations, trends, and patterns described here do not necessarily imply causality, and the lack of information on spatial patterns prevents inferences at lower scales (such as neighborhoods). Furthermore, the interaction between social, economic, and environmental factors and changes in access to healthcare or the availability of diagnostic tests may influence the ability to diagnose cases^14^ ^,^ ^15^. In addition, antigenic variations in the dominant serotype or the introduction of new dengue virus genotypes/serotypes, co-circulation of other arboviruses such as *Zika* or *Chikungunya*, and viral competition between viruses transmitted by *Aedes* species could alter endemicity and cause an increase in febrile cases (with and without laboratory diagnosis) during certain periods^1^ ^,^ ^14^. Furthermore, dengue incidence is also influenced by dengue-specific population immunity to circulating serotypes and even cross-protective immunity to other *flaviviruses* ^1^. None of these factors were analyzed in this ecological study. Despite limitations related to the design and analysis and the likely reporting bias because we analyzed data from the surveillance system, which mainly collects information on symptomatic cases, we believe that our results provide useful information for the implementation and strengthening of dengue prevention and control strategies in Cartagena. 

In conclusion, we described the relationships between climatic variables and dengue case incidence in Cartagena, Colombia. Temperature, humidity, precipitation, and rainy days were significantly correlated with dengue case occurrence in non-lag or lagged months, while macroclimatic phenomena did not appear to play an important role in the dynamics of the disease in the city of Cartagena. Additionally, our findings suggest that dengue is not seasonal in the city. Hence, prevention efforts need to be constant throughout the year, given that Cartagena is a tropical and dengue-endemic area.
